# The utility of self-rated health in population surveys: the role of bodyweight

**DOI:** 10.1186/s12963-021-00255-2

**Published:** 2021-05-03

**Authors:** Robert Bozick

**Affiliations:** Kinder Institute for Urban Research, Rice University, Kraft Hall, 6100 Main Street, Suite 305, Houston, TX 77005-1892 USA

**Keywords:** Self-rated health, Survey research, Obesity, Bodyweight

## Abstract

**Background:**

Self-rated health (SRH) is one of the most commonly used summary measures of overall health and well-being available to population scientists due to its ease of administration in large-scale surveys and to its efficacy in predicting mortality. This paper assesses the extent to which SRH is affected by its placement before or after questions about bodyweight on a survey, and whether differences in placement on the questionnaire affects SRH’s predictive validity.

**Methods:**

I assessed the validity of SRH in predicting the risk of mortality by comparing outcomes of sample members who were asked to rate their health before reporting on their bodyweight (the control group) and sample members who were asked to rate their health after reporting on their bodyweight (the treatment group). Both the control and treatment group were randomly assigned via an experiment administered as a module in a nationally representative sample of adults in the USA in 2019 (*N* = 2523).

**Results:**

The odds of reporting a more favorable appraisal of health are 30% lower for sample members who were in the treatment group when compared with the control group. Additionally, the SRH of treatment group members is significantly associated with their risk of mortality, while the SRH of control group members is not.

**Conclusion:**

The findings from this study suggest that for researchers to maximize the utility of SRH, closer attention needs to be paid to the context of the survey within which it asked. SRH is highly sensitive to the questions that precede it, and this sensitivity may in turn mischaracterize the true health of the population that the survey is intending to measure.

## The utility of self-rated health in population surveys: the role of bodyweight

Self-rated health (SRH) is one of the most commonly used summary measures of overall health and well-being available to population scientists. Its widespread use is partially due to its ease of administration in large-scale surveys and partially due to its efficacy in predicting key demographic outcomes—namely mortality [2, 8, 11, 19, 21]. Further, it permits efficient comparisons of the overall health of populations that may differ in the particulars of their environments and corresponding health risks. In an era of increasing competition for the time and attention of sample members alongside declining response rates [7], survey developers are tasked with the challenge of minimizing the burden of answering a questionnaire while simultaneously maximizing the value of the information collected. SRH has emerged as a critical survey item because it serves both ends.

SRH is a low-burden item, typically asked in a concise, straight-forward manner: “In general, how do you describe your overall health? Excellent, very good, good, fair, or poor?” Despite its brevity, information gleaned from the responses to this question has yielded a wealth of information by characterizing an array of key demographic relationships at the population level. Individuals who report being in excellent or very good health tend to have stronger immune systems [5], lower levels of allostatic load [31], lower rates of depression [1], and a lower risk of chronic disease and disability [14]. Additionally, individuals who report being in excellent or very good health significantly contribute to key demographic behaviors, as evidenced by higher rates of migration [10], marriage [27], and fertility [24]. With so much riding on a single survey item, researchers have sought to probe further into the measurement properties of SRH to better understand how sample members interpret and subsequently respond to the question. Such methodological work has included assessing mode effects, question context (i.e., adjacent items), response option labels, response option order, and language of the interview [3, 15, 16, 25, 32].

In this study, I contribute to this growing methodological research base on the utility of SRH by examining how responses to SRH are explicitly or inadvertently affected by other dimensions of health that are directly asked of sample members within the same survey. I focus on a single but important contributor to overall health and well-being: bodyweight. Self-reports of bodyweight are increasingly included in population health surveys because they are used to gauge the prevalence and consequences of obesity, which currently affects 39.8% of the adult population in the USA [17]. Specifically, I will test the hypothesis that sample members downwardly adjust their SRH if they are first asked to report on their bodyweight. Next, I perform a hypothetical predictive validity exercise to test the hypothesis that these “bodyweight primed” measures of SRH are better predictors of the risk of mortality than “bodyweight agnostic” measures of SRH. In what follows, I first review research on how SRH is affected by properties of survey instruments as a contextual foundation for these two hypotheses. I will then test these two hypotheses with data from a randomized experiment conducted using a nationally representative survey of adults in the USA. I conclude with a discussion on the implications of my findings for both survey developers and population health scientists.

## Background

Despite its widespread use and ease of administration, SRH remains a “volatile survey item” because how sample members respond to this survey item is directly affected by the properties of the survey in which it is asked [15]. To date, the research base suggests that this volatility is largely driven by the order of response options and on the placement of the SRH item in the context of other survey items. While not dismissing the importance of the former, this paper specifically contributes to the growing body of research on the latter.^1^ In a handful of studies, researchers have documented that when sample members are asked to rate their health *after* a series of items about specific health conditions, they are more likely to report being in poor health than if they were asked to rate their health prior to the same health condition items [6, 16]. This comports with theories about cognitive priming in psychology which posit that exposure to one stimulus (in this case, questions about the prevalence of personal health conditions) influences a response to a subsequent stimulus (in this case, a question about SRH) without conscious guidance or intention [22]. Put simply, if sample members are prompted to think about specific health conditions they may have as they fill out a survey—even if only briefly—those conditions may unconsciously inform how they appraise their own health when asked a more generic item such as SRH.^2^

The studies showing evidence of this priming effect are based on surveys where antecedent questions assumed to be affecting responses are about specific health conditions such as the presence of asthma or diabetes or health behaviors such as exercising and smoking cigarettes [6, 16, 26]. These questions draw attention to dimensions of health that are in general, easy to diagnose and easy to understand by the sample member. Further, health conditions like asthma or diabetes typically require medication schedules, interactions with doctors, and other forms of ongoing care—even if the condition is mild. Consequently, they have seemingly straightforward connections to health and by extension, individual perceptions of their own health that likely affect how they might respond to a SRH question on a survey.

In this analysis, I extend the work of previous researchers who find evidence of a priming effect when asked to rate their own health. However, instead of including a disparate array of health conditions and health behaviors, I focus on a single dimension of health that may be less obvious to sample members as a primary health indicator but potentially a greater risk factor for mortality compared with more mild health conditions that are often collected in surveys: bodyweight. Over the past couple of decades, bodyweight has emerged as an important measure for population health scientists both because of the increase in the prevalence of obesity [17] and because of obesity’s strong relationship with morbidity and mortality [12, 28]. Affecting over one third of the adult population in the USA [17], obesity is the second leading preventable cause of death behind tobacco use [12]. Obesity is of particular relevance for self-assessments of overall health as individuals make daily choices about their food intake and physical activity that in turn cumulatively shape their physical and mental health. Because food intake and physical activity are so deeply entrenched into daily life functions and routines, they may not cognitively register as critical inputs when individuals are asked on surveys to assess their overall health.

Obesity is a particularly thorny health condition to ask about in large scale surveys as nearly half of obese individuals do not know or believe they are obese [33]. This lack of awareness is exacerbated by doctors underdiagnosing obesity because they are uncomfortable discussing bodyweight issues with their patients [23]. The implication here is that while obesity severely impedes long-term health and wellbeing, one’s own bodyweight might fail to register as a consideration when determining an overall rating of personal health status. If individuals ignore their bodyweight and/or are unsure of their obesity status when filling out surveys, it could induce substantial measurement error that may attenuate the utility of SRH as a reliable indicator of one’s health status. Put differently, if sample members are not considering the most critical indicators of their health when answering the question “In general, how do you describe your overall health?” then their responses may be less valuable in predicting demographic and health outcomes.

In this study, I examine the potential role of bodyweight in shaping the utility of SRH by building off a novel research study undertaken by Lee and Schwarz [26]. In their study, they compared mortality rates of elderly sample members who were asked SRH before a series of health conditions (using data from the Health and Retirement Study) with mortality rates of elderly sample members who were asked SRH after a series of questions about health conditions (using data from the National Health Interview Survey). They found that among Spanish-speaking sample members, the relationship between SRH and mortality was stronger in the National Health Interview Survey, where SRH was asked in the context of other health conditions. This suggests that the utility of SRH to predict mortality depends on the design of the questionnaire in which it is asked.

While innovative and informative, Lee and Schwarz’s [26] study used two different, independent surveys collected by different agencies, and so it is not clear if the observed mortality differences are due to the placement of SRH in the context of other health conditions or due to differences in survey design, sampling properties, the populations surveyed, and/or data collection procedures of the two studies. Further, in focusing only on the elderly, who have substantially higher rates of morbidity than the general adult population and who are closer in time to their own mortality, the generalizability of their findings toward the younger adult population, who face different health risks and for whom mortality is a more distal event, is unclear. I build upon their work by comparing differences in responses within the same survey, thereby mitigating other confounding factors that may emerge when comparing response from two independent surveys. Additionally, I use a sample of the full adult population in the USA, and so any potential age-specific appraisals of health that might be particular to the elderly are minimized.

## Methods

### Study design

This study uses data from the RAND Corporation’s American Life Panel (ALP), a nationally sampled online panel that permits generalization to the non-institutionalized population of adults in the USA. Since its inception in 2003, the panel receives a standard module on household characteristics every quarter as well as periodic surveys on different topics throughout the year. For this analysis, I used a set of questions fielded as part of an experiment included in an ALP omnibus survey that was administered to sample members between February 20, 2019, and April 7, 2019. Respondents participated online, either using their own devices or via RAND-provided internet access. With a single mode of data collection, any potential mode effects are eliminated. For more details about the ALP, see Pollard and Baird [29].

For this omnibus survey, 3932 ALP members aged 21 and older were invited to participate, with the goal of obtaining at least 2500 responses. The survey was administered in English only. Invited participants were randomly selected from English-speaking, probability-based active panel members (defined as those who completed a survey within the past year). Over the 6-week period when the survey was fielded, 2555 responded, yielding a response rate of 64.9%. Of those 2555 respondents, I eliminated 30 sample members who were 85 years of age and older so as to remove any confounding effects of bodyweight owing to increased frailty among the elderly. I then eliminated one sample member who did not provide a response to the SRH question and another sample who did not provide their bodyweight. The final analytic sample includes 2523 respondents.

With these data, I test two distinct hypotheses. First (*H*_*1*_), sample members will downwardly adjust their SRH if they are first asked to report on their bodyweight. Second (*H*_*2*_), SRH will have more utility in predicting the risk of mortality when it is preceded by questions about bodyweight. To permit an examination of these two hypotheses, ALP sample members were randomly assigned to one of two conditions, which are shown in more detail in Table 1. The control group was asked to rate their overall health first, then answer questions about their height, weight, and perceptions of their bodyweight. The treatment group was first asked about their height, weight, and perceptions of their bodyweight, before being asked to rate their overall health.

**Table 1:** Question order differences for the control and treatment groups

Control Group	Treatment Group
Question 1	Question 1
How do you describe your overall health in general?	How tall are you (feet/inches) without shoes?
Excellent	Question 2
Very good	How much do you weigh (in lbs) without clothes or shoes?
Good	Question 3
Fair	How do you think of yourself?
Poor	Very underweight
Question 2	Slightly underweight
How tall are you (feet/inches) without shoes?	About the right weight
Question 3	Slightly overweight
How much do you weigh (in lbs) without clothes or shoes?	Very overweight
Question 4	Question 4
How do you think of yourself?	How do you describe your overall health in general?
Very underweight	Excellent
Slightly underweight	Very good
About the right weight	Good
Slightly overweight	Fair
Very overweight	Poor

The randomization yielded balance, such that the control group (*n* = 1264) and the treatment group (*n* = 1259) are similar on key observed demographic characteristics, as shown in Table 2. There are comparable percentages across the two groups with respect to their sex, race/ethnicity, age, and education level. Note that for ease of presentation, I collapsed ages into broad generational categories used to define American age cohorts as defined by the Pew Research Center [9], such that at the time of the survey millennials were between the ages of 22 and 38, generation X was between the ages of 39 and 54, baby boomers were between the ages of 55 and 73, and the silent generation was 74 and older.

**Table 2:** Characteristics of the control and treatment groups. Data are from the RAND Corporation’s American Life Panel, February–April 2019

	Control Group		Treatment Group	
	%	n	%	n
Sex				
Female	56.9	719	56.9	717
Male	43.1	545	43.1	542
Race/ethnicity				
Black	9.7	122	9.8	124
Hispanic	14.4	182	15.2	191
Other	4.9	62	5.0	63
White	71.0	898	70.0	881
Age				
Millenial: Age 22 - 38	12.5	158	14.3	180
Generation X: Age 39 - 54	24.1	304	26.0	327
Baby boomer: Age 55 - 73	52.6	665	49.5	624
Silent generation: Age 75+	10.8	137	10.2	128
Education level				
High school or less	14.7	186	13.4	169
Some college or an associate's degree	34.3	433	35.8	451
Bachelor's degree	26.3	333	27.5	346
Graduate/professional degree	24.7	312	23.3	293
Body mass index				
Underweight	2.1	27	1.5	19
Healthy weight	24.6	311	27.4	345
Overweight	34.0	430	33.4	420
Obese	39.3	496	37.7	475
Bodyweight perception				
Very underweight	0.8	10	1.2	15
Slightly underweight	4.5	57	5.5	69
About the right weight	23.5	297	24.3	306
Slightly overweight	44.4	561	44.6	562
Very overweight	26.8	339	24.4	307
N		1,264		1,259

Using sample members’ self-reported height and weight, I first converted their responses to the metric system and then calculated their body mass index (BMI) by dividing their weight in kilograms by their height in meters squared. Using cut points for adults prescribed by the U.S. Department of Health and Human Services, I used this continuous measure to classify sample members as underweight (BMI < 18.5), healthy weight (BMI between 18.5 and 24.9), overweight (BMI between 25 and 29.9), and obese (BMI ≥ 30). The modal category for both groups is obese. Lastly, the distribution of bodyweight perceptions is nearly identical across both groups, with feeling “slightly overweight” as the mode.^3^ That we find similarities across demographic characteristics and across measures of bodyweight provides further confidence that the randomization was effective in minimizing group differences.

### Empirical approach

To test *H*_*1*_, I estimate an ordered logit model predicting the ordinal measure of SRH, reverse coded such that higher values indicate better health (i.e., 5 = excellent,…1 = poor). The key predictor of interest is a binary variable coded “1” if the sample member was assigned to the treatment group and “0” of the sample member was assigned to the control group. The associated parameter for this binary variable will indicate whether or not the treatment group downgrades their SRH relative to the control group. Even though the treatment and control groups are balanced on demographic characteristics and bodyweight measures, I also include these as control variables in the model—thus producing “doubly robust” parameter estimates [13]. Note that the observed treatment effects are unaffected by the inclusion or exclusion of these additional controls.

To test *H*_*2*_, I perform a hypothetical predictive validity exercise. One of the key steps for psychometricians in evaluating the measurement properties of key metrics is to assess how well the metric of interest predicts an outcome (or “criterion”) for which it should theoretically have a strong association. If the metric (observed at time *t*) is strongly associated with the outcome (observed at *t* + *x*, where *x* is a sufficient period of time), the measure is considered to have predictive criterion validity. The ideal assessment of SRH’s predictive validity would involve longitudinally following ALP sample members for a long enough period of time after the omnibus survey when SRH was measured to observe patterns of mortality among individual sample members. With such data, I would be able to ascertain the longitudinal relationship between SRH (at *t*) and the most vital health criterion, mortality (at *t* + *x*). Given the impracticality of that design with the limited cross-sectional data on adults who are currently at ages with relatively low mortality rates, I instead perform a hypothetical predictive validity exercise in which I use mortality data from the U.S. Department of Health and Human Services’ Center for Disease Control to create a proxy measure of sample members’ *predicted risk of death*.

Although mortality rate data from 2019 would be preferred, 2017 is the most recently available data at the time of this analysis. Therefore, I used observed mortality rates for 2014, 2015, 2016, and 2017 to linearly extrapolate values for 2018 and for 2019. To eliminate fluctuations in rates over time, which can be exacerbated when calculated for small areas such as counties, I took the mean of these six values (where four were observed and two were extrapolated). I use this “smoothed” rate as the dependent variable. Sensitivity analyses (not shown) reveal that the results are similar if instead I use the most recent observed county-level mortality rate (2017) as the dependent variable or if I use the most recent extrapolated county-level mortality rate (2019) as the dependent variable.

These county-level rates, which are standardized using the 2000 U.S. standard population, are assigned as the outcome for the sample member contingent on their own race, sex, and county of residence. Rates at the county level are only calculated for Blacks and Whites due to small cell counts for other racial/ethnic groups, and so I restrict this portion of the analysis to Black and White sample members only. I use negative binomial regression to estimate the relationship between sample members’ SRH and their predicted risk of death, separately for those in the control group and for those in the treatment group. I then compare the parameter estimates associated with SRH across both models, with the expectation that SRH will be a better predictor of the risk of mortality among the treatment group. As with the ordered logit model testing *H*_*1*_, I include controls for demographic characteristics, BMI, and perceptions of bodyweight in these models.

## Results

**Fig. 1:**
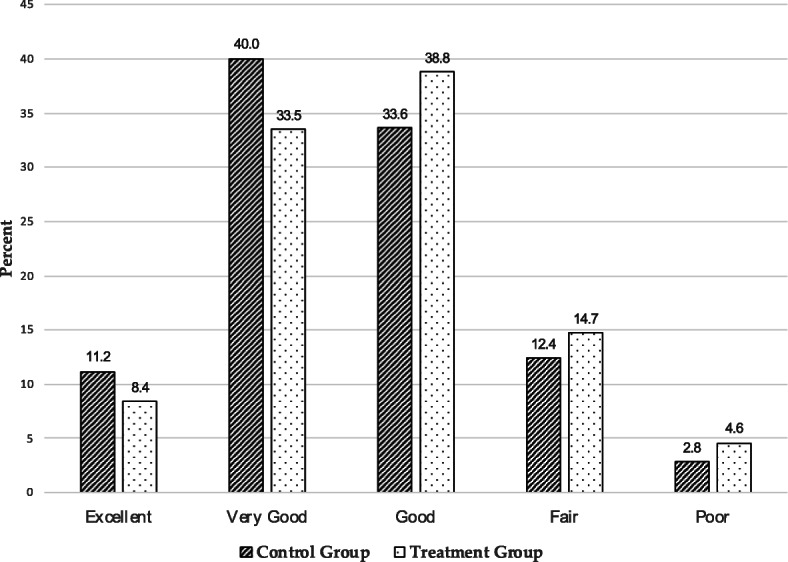
Levels of self-rated health by experimental condition. Data are from the RAND Corporation’s American Life Panel, February–April 2019

The first analytical task is to assess the evidence in support of the hypothesis that that sample members will downwardly adjust their SRH if they are first asked to report on their bodyweight (*H*_*1*_). Before showing multivariate results, I first show the unadjusted, univariate distributions of SRH as reported by the control and treatment groups in Fig. 1. As expected, control group members, who were asked to rate their health *before* answering questions about their bodyweight, reported being in excellent or in very good health at higher rates when compared with treatment group members. Conversely, treatment group members, who were asked to rate their health *after* answering questions about their bodyweight, reported being in good, fair, or poor health at higher rates when compared with control group members. Group differences are largest among those who report being in very good health: 40.0% of the control group vs. 33.5% of the treatment group. While the overall differences between the two groups are not particularly large, ranging from 1.8% (among those who rated their health as poor) to 6.5% (among those who rated their health as very good health), it is worth remembering that these two groups—which were developed by random assignment—have nearly identical demographic and bodyweight profiles. The only observable difference between the two groups is the order of the questions on the survey per the experimental conditions shown in Table 1.

To assess whether this relationship holds in a multivariate context, I use an ordered logit model to predict the five levels of SRH as a function of treatment/control group membership, demographic characteristics, and bodyweight measures. Unbiased estimates from an ordered logit model requires fulfillment of the proportional odds assumption, which assumes that the slope estimate for our key variable of interest (i.e., the question order experiment indicator) between each pair of outcomes across two adjacent levels of SRH is the same regardless of which two adjacent levels of SRH are considered. A non-significant Brant test statistic (*χ*^2^ = 2.11, *p* = 0.55) indicates this assumption has been met [4]. However, it should be noted that the results are robust to model specification. For example, I treated SRH as continuous and estimated an OLS model and I treated SRH as binary (where excellent and very good health = 1; good, fair, and poor health = 0) and estimated a standard logit model. The results (not shown) are comparable regardless of what estimation function is used. I show the results for the ordered logit model because they most accurately reflect the underlying distribution of an ordered categorical outcome. I present odds ratios from this ordered logit model along with their 95% confidence intervals in Table 3. In ordered logit models, odds ratios can be interpreted as follows: for a one-unit increase in the explanatory variable *x*_*k*_, the odds of a lower value of SRH compared with a higher value of SRH are changed by the factor of exp(−*β*_*k*_), holding all other variables in the model constant.

**Table 3:** Odds ratios from an ordered logit model predicting self-rated health. Data are from the RAND Corporation’s American Life Panel, February–April 2019

	Odds Ratio	95% Confidence Interval
Question order experiment		
Treatment group	0.70^**^	(0.61, 0.81)
Control group (reference)	1.00	__
Sex		
Female	1.23^**^	(1.05, 1.43)
Male (reference)	1.00	__
Race/Ethnicity		
Black	0.63^**^	(0.49, 0.81)
Hispanic	0.70^**^	(0.56, 0.87)
Other	0.69^*^	(0.49, 0.98)
White (reference)	1.00	__
Age		
Millenial: Age 22 - 38 (reference)	1.00	__
Generation X: Age 39 - 54	0.82	(0.64, 1.05)
Baby boomer: Age 55 - 73	0.85	(0.67, 1.07)
Silent generation: Age 75+	0.70^*^	(0.51, 0.96)
Education level		
High school or less (reference)	1.00	__
Some college or an associate's degree	1.35^*^	(1.07, 1.70)
Bachelor's degree	2.01^**^	(1.57, 2.57)
Graduate/professional degree	2.32^**^	(1.80, 2.98)
Body mass index		
Underweight	0.60	(0.34, 1.05)
Healthy weight (reference)	1.00	__
Overweight	0.99	(0.80, 1.23)
Obese	0.70^**^	(0.54, 0.90)
Bodyweight perception		
Very underweight	0.14^**^	(0.06, 0.31)
Slightly underweight	0.60^**^	(0.42, 0.86)
About the right weight (reference)	1.00	__
Slightly overweight	0.58^**^	(0.47, 0.73)
Very overweight	0.23^**^	(0.17, 0.31)
N	2,523	

Our key parameter of interest in Table 3 is the one that corresponds with the experimental condition. The estimated odds ratio is 0.70 and is statistically significant at *p* < 0.01. This indicates that the odds of reporting a more favorable appraisal of health are 30% lower for sample members who were first asked questions about their bodyweight. This provides support for (*H*_*1*_), which states that sample members will downwardly adjust their SRH if they are first asked to report on their bodyweight. I speculate this is the case because the suggestion of bodyweight, which is a critical input to overall health, cognitively primes sample members such that they are more likely to take their bodyweight into consideration when asked to rate their health.

Although we are most interested in the parameter estimate for the experimental condition, it is worth pointing out that the two measures of bodyweight—one objective and one subjective—are both significant predictors of SRH. Those who are obese per their BMI are more likely to report being in worse health than their peers who are a healthy weight. Additionally, those who think they are over- or under-weight are more likely to report being in worse health than those who think they are about the right weight. This aligns with other research which finds evidence of a strong correlation between bodyweight and SRH [20, 30].

The second analytical task is to assess the evidence in support of the hypothesis that SRH will have more utility in predicting the risk of mortality when it is preceded by questions about bodyweight (*H*_*2*_). As we saw in the ordered logit models, SRH appears to be distinctively affected when sample members are first prompted to report their bodyweight and their perceptions of their bodyweight. Therefore, I consider the treatment group to have “bodyweight primed” measures of SRH and the control group to have “bodyweight agnostic” measures of SRH. Given that “bodyweight primed” measures of SRH are imbued with information about one’s own obesity status, which is an important predictor of the onset of high blood pressure, diabetes, heart disease, and stroke, as well as subsequent mortality associated with these conditions, I anticipate that they will be stronger predictors of the risk of mortality than “bodyweight agnostic” measures of SRH. I test this hypothesis in a series of negative binomial regression models shown in Table 4.

**Table 4:** Coefficients from negative binomial regression models predicting the estimated risk of death for sample members based on their sex, race, and county of residence. Data are from the U.S. Department of Health and Human Services’ Center for Disease Control and the RAND Corporation’s American Life Panel, February–April 2019

	Control Group		Treatment Group	
	Coefficient	95% Confidence Interval	Coefficient	95% Confidence Interval
Self-rated health	-0.001	(-0.014, 0.012 )	-0.019^**^	(-0.031, -0.007)
Sex				
Female	-0.298^**^	(-0.322, -0.274)	-0.307^**^	(-0.329, -0.285)
Male (reference)	__	__	__	__
Race				
Black	0.279^**^	(0.243, 0.315)	0.281^**^	(0.246, 0.315)
White (reference)	__	__	__	__
Age				
Millenial: Age 22 - 38 (reference)	__	__	__	__
Generation X: Age 39 - 54	-0.018	(-0.060, 0.025)	0.006	(-0.37, 0.050)
Baby boomer: Age 55 - 73	-0.019	(-0.058, 0.021)	0.019	(-0.021, 0.059)
Silent generation: Age 75+	-0.025	(-0.075, 0.024)	0.014	(-0.034, 0.062)
Education level				
High school or less (reference)	__	__	__	__
Some college or an associate's degree	0.003	(-0.035, 0.041)	0.000	(-0.035, 0.035)
Bachelor's degree	-0.038	(-0.077, 0.002)	-0.025	(-0.061, 0.012)
Graduate/professional degree	-0.048^*^	(-0.088, -0.007)	-0.029	(-0.066, 0.008)
Body mass index				
Underweight	0.031	(-0.065, 0.126)	-0.063	(-0.146, 0.019)
Healthy weight (reference)	__	__	__	__
Overweight	0.018	(-0.014, 0.050)	-0.000	(-0.033, 0.032)
Obese	0.034	(-0.004, 0.073)	0.024	(-0.014, 0.063)
Bodyweight perception				
Very underweight	-0.034	(-0.146, 0.077)	0.117	(-0.021, 0.254)
Slightly underweight	-0.008	(-0.064, 0.048)	-0.002	(-0.059, 0.055)
About the right weight (reference)	__	__	__	__
Slightly overweight	-0.003	(-0.035, 0.029)	0.008	(-0.025, 0.040)
Very overweight	-0.03	(-0.076, 0.015)	-0.012	(-0.056, 0.032)
Likelihood-Ratio χ^2^	715.48		842.85	
N	1,003		1,015	

In the model, the outcome is the age-adjusted county-level mortality rate (per 100,000 persons) assigned to the sample member based on their race, sex, and county of residence. As described earlier, this outcome serves as a proxy of the risk of death for each sample member. The model includes the five-level ordinal measure of SRH along with controls for demographic characteristics and bodyweight measures. The left panel of Table 4 shows the parameter estimates from a model estimated using the control group sample and the right panel of Table 4 shows the parameter estimates from a model estimated using the treatment group sample.

In the left panel, the parameter estimate for SRH among control group members is negative (*β* = −0.001) but does not reach the threshold for statistical significance. In the right panel, the parameter estimate for SRH among treatment group members is also negative but larger in magnitude (*β* = −0.019). However, unlike for the control group, the parameter estimate reaches the threshold for statistical significance at *p* < 0.01. The literal interpretation of this significant coefficient is “each level increase of the five levels of SRH for a sample member is associated a decrease of 0.019 in the log count of deaths within a county among individuals of the same sex and same race as the sample member.” In simpler terms, this means that SRH is associated with the risk of dying, such that those who report being in excellent health have a lower risk of death while those who report being in poor health have a higher risk of death.

That I observe a significant relationship between SRH and the predicted risk of mortality among the treatment group but not the control group provides evidence in support of the hypothesis that “bodyweight primed” measures of SRH have stronger predictive validity than “bodyweight agnostic” measures of SRH. This is further buttressed by the improvement in the likelihood-ratio chi-square in the model estimated using the treatment group (*χ*^2^= 842.85) compared with the likelihood-ratio chi-square in the model estimated using the control group (*χ*^2^= 715.48).

## Discussion

This paper contributes to the growing methodological research base on the administration and utility of self-rated health (SRH) in population-based surveys by examining how responses to SRH are explicitly or inadvertently affected by other dimensions of health that are directly asked of sample members within the same survey. I focus on a single dimension of health that is correlated with a host of short- and long-term health issues: bodyweight. To do so, I conducted an experiment in the context of a nationally representative survey of adults in the USA. My analysis yielded two findings of note.

First, I find that when filling out surveys, sample members who are asked questions about their bodyweight before being asked to rate their own health were more likely to report being in worse health than sample members who were asked to rate their health before questions about their bodyweight. The magnitude of these differences comports with other research which finds similar patterns when toggling the order of questions [6, 16], and is in alignment with theories about cognitive priming in psychology which suggests that exposure to one stimulus influences a response to a subsequent stimulus without conscious guidance or intention [22]. However, unlike other studies which included anywhere from eight questions [16] to 36 questions [6] encompassing an array of health conditions and behaviors before asking SRH, the present study only asked about bodyweight. That I observe a significant reduction in overall health when SRH is asked after inquiring about a single dimension of health indicates that SRH is highly sensitive to where it is placed on a survey instrument.

Second, I find that this sensitivity has consequences for the utility of the resulting data collected. When sample members are cognitively primed to consider their own bodyweight prior to rating their health, the validity of the resulting rating in predicting their own estimated risk of mortality is improved. Conversely, sample members who are asked to rate their health at the start of the survey, before being asked questions about their bodyweight, yield measures of SRH that are unrelated with their expected risk of mortality. This suggests that the placement of SRH on surveys is critical for how useful the resulting responses are as a gauge for long-term health and well-being. My results, based on an experiment within a single survey administered to nationally representative sample of adults, corroborate similar patterns observed in independent surveys administered only to the elderly [26].

While my study has a number of strengths, including a randomized experiment with national-level generalizability, the findings should be considered in the context of two key limitations. First, self-reports of height and weight are less precise than direct anthropometric measurements. However, there is a growing empirical consensus that self-reports are a reliable substitute in large-scale surveys where anthropometric measurement is not possible (see [18]). Second, because the analysis was based on cross-sectional data, it was not possible to do a traditional assessment of SRH’s predictive validity using observed mortality as a criterion observed at a later date. Instead, I used race and sex-specific county-level rates of mortality as a proxy measure of sample members’ *predicted risk of death* and conducted a hypothetical predictive validity exercise. Because I use predicted rather than observed mortality, these findings should be treated as suggestive rather than definitive.

These limitations notwithstanding, the findings from this analysis have important implications for both survey developers and for researchers. Whenever possible, survey developers should consider placing SRH after items that require sample members to report on health conditions, as doing so improves the utility of SRH. Many studies which attempt to efficiently quantify overall health via SRH will use it apart from measures of other health conditions, often as the only measure of health in a statistical model. This places undue responsibility on SRH to be an all-encompassing, multi-purpose item. Therefore, the more SRH can be informed by dimensions of health that are most consequential for long-term well-being, the more useful it will be as a stand-alone measure. Surveys where health is not the primary focus and/or surveys with constraints on the number of questions that can be included often cannot accommodate a battery of health measures (such as bodyweight) prior to asking SRH. In these instances, survey developers may want to consider providing instructions to sample members to prompt them to initiate a more thorough internal accounting of conditions and behaviors that contribute to their true health status.

Researchers that include SRH in their analyses should whenever possible review the content and structure of the questionnaires used to collect the data. Where it makes sense to do so, such information should be included when describing the construction of the measure for the analysis at hand. Doing so will provide context that can help the reader interpret the results. This is particularly important when SRH is asked before a series of health conditions or when SRH is the only health measure on the survey. In the USA, the major population-based longitudinal surveys used to gauge life course development and health—including the National Longitudinal Study of Adolescent to Adult Health, the National Longitudinal Survey of Youth, and the Health and Retirement Study—all measure SRH before asking any specific questions about health conditions and behaviors. This is also the case in major international surveys such as the Australian National Health Survey, the Canadian Health Measure Survey, the Health Survey for England, the European Health Interview Survey, the Spain National Health Survey, as well as the multi-nation surveys administered as part of the Demographic and Health Surveys Program. In contrast, the German Health Interview and Examination Survey and the United States’ National Health Interview Survey measure SRH after asking about a series of health conditions. In analyses that produce null SRH effects using survey data where SRH precedes questions about health conditions, researchers should not immediately conclude that health status has no effect. This null finding could be due to the design of the survey rather than a lack of a true health effect in the larger population.

## Conclusion

In closing, SRH will likely remain a popular item to include on population health surveys for decades to come. The findings from this study suggest that for researchers to maximize the utility of this item moving forward, closer attention needs to be paid to the context of the survey within which it asked. SRH is highly sensitive to the questions that do or do not precede it, and this sensitivity may in turn mischaracterize the true health of the population that the survey is intending to measure.

## Data Availability

Data used for this study are publicly available at https://alpdata.rand.org/.

## Abbreviations

ALP: American Life Panel
BMI: Body mass index
SRH: Self-rated health

## Footnotes

1 In sum, the evidence to date suggests that individuals are more likely to appraise their health favorably if the response options are ordered from “Excellent” to “Poor” and less likely to appraise their health favorably if the responses options are ordered from “Poor” to “Excellent” (see [16]).

2 It is worth noting that not all methodological work finds evidence of this priming effect. For example, Lee and Grant [25] administered the 2007 California Health Interview Survey with half of the sample asked SRH before a series of questions about health conditions and the other half asked SRH after a series of questions about health conditions. When the survey was administered in Spanish, SRH evidenced poorer health if asked before the series of questions about health conditions.

3 It is interesting to note that the modal category of BMI is “obese,” yet the modal category of bodyweight perceptions is “slightly overweight.” This discrepancy further underscores the research cited earlier that nearly half of obese individuals do not know or believe they are obese [33].
